# Urban-Rural Differences of Impact of the Accessibility of Community Older Adult Care Services on the Quality of Life of Older Adults

**DOI:** 10.3389/ijph.2026.1609432

**Published:** 2026-03-26

**Authors:** Xiaodong Di, Lijian Wang

**Affiliations:** School of Public Policy and Administration, Xi’an Jiaotong University, Xi’an, China

**Keywords:** accessibility, influence mechanism, quality of life of older adults, social support, urban-rural differences

## Abstract

**Objectives:**

This study aimed to balance the resources for older adult care services in urban and rural areas, improve the quality of older adult care services, and enhance the quality of life for older adults.

**Methods:**

Based on the survey data of Shaanxi province and the ordinal logistic regression method, the paper analyzes the index system and measurement results of the accessibility of community older adult care services, and explores the urban-rural differences of impact of accessibility on the quality of life of older adults from the perspective of social support.

**Results:**

The accommodation of community older adult care service has a significant positive impact on the social relationship of urban older adults, while the five dimensions of the accessibility of older adult care services have no significant impact on the social relationship of rural older adults.

**Conclusion:**

The urban-rural differences of the impact are mainly reflected in the two aspects of accommodation and affordability. Government support and social organization support are the main reasons for the heterogeneity.

## Introduction

Integrating the basic public service system and enhancing the balance and accessibility of public services are important strategies for coping with population ageing. By the end of 2024, there were 8.87 million people over the age of 60, accounting for 22.4 percent of Shaanxi province’s population, 6.20 million people over the age of 65 and over, accounting for 15.7 percen. It takes Shaanxi 20 years to transition from an aging society to a moderately aged society, which is much faster than that of developed countries such as the United States and Japan. However, during the same period, the *per capita* GDP just exceeds 10,000 US dollars [1]. However, due to the problem of urban-rural dual sector model in Shaanxi for a long time [2, 3], the differences in economic development level, public facilities supply, and community older adult care culture make the urban-rural differences in community older adult care services particularly prominent. In rural areas of Shaanxi, due to the wide distribution of older adult, backward economic development, weak public services and limited older adult care facilities, older adults’s demand for community older adult care services is more focused on family care and medical care [4]. In urban areas, because there are sufficient elements such as information, technology, capital and personnel, older adults can choose more contents and forms of older adult care services, pay more attention to the accessibility and effectiveness of community older adult care services, and prefer emotional comfort, social communication and other needs [5]. The differences lead to such practical problems as the imbalance allocation of older adult care resource, the low utilization rate of service facilities, and the mismatch between service supply and demand in older adult care contents [6–8], which seriously limits the quality of life of older adults. Therefore, from the perspective of the integrated development of urban and rural older adult care services, improving the quality of community older adult care services has become an important livelihood issue to reflect the people-oriented development concept and meet the diversified needs of the people’s older adult care services.

Existing literatures on the differences between urban and rural community older adult care services mainly focus on two aspects. On the one hand, some literatures focus on the reasons of the differences. The first reason is there is difference in the degree of aging in different areas. According to the press conference on the development status of older adult affairs in Shaanxi Province in 2024, the city with the highest aging rate, Baoji City (29.21%), differs by 14.9 percentage points from the city with the lowest rate, Yangling Demonstration Zone (14.31%), reflecting the imbalance in regional development. Colibaba et al also thought rural communities worldwide were experiencing the most significant levels of aging in place, and remote areas had a larger proportion of older people [9]. The second reason is the difference of economic development level between urban and rural areas in Shaanxi. In 2024, the *per capita* disposable income of urban residents is 46,821 yuan, and that of rural residents is 18,199 yuan [10], indicating a significant gap between urban and rural income. And Giannakis from the perspective of economic resilience, found large urban centres, especially those hosting diversified high-value functions, were more resilient to recession than towns and rural areas [11]. The third reason is the difference of infrastructural facilities between urban and rural areas. By the end of 2025, the coverage rate of urban day care centers in Shaanxi Province reaches 93.8%, while the coverage rate of rural mutual aid nursing home only reaches 82% in rural villages. [12], so service facilities for older adults is limited in rural areas. Cheng also found the inequity in accessibility displays an increasing trend from the city centre outwards by analysing the geographical accessibility to hospitals specific to older adults population in Nanjing, China [13]. On the other hand, some literatures focus on the impact of the differences of older adult care services on the quality of life of older adults, including the impact of care content on the health of older adults [14], the impact of pension on the quality of life of older adults [15], and the welfare effect of care resources [16]. On the whole, the first type of literatures focuses on the objective differences between urban and rural community older adult care services, ignoring the impact of social support on the differences of older adult care services, while studying the impact of different service providers on community older adult care services can effectively improve the allocation and organization of older adult care resources and improve the utilization rate of care resources. The second type of literatures focuses on the whether a certain service content has an impact on the quality of life of older adults. It analyzes what content should be supplied in the community older adult care service from the perspective of quantity, and rarely analyzes how to improve community older adult care service from the perspective of quality.

Therefore, in order to balance older adults care resources in urban and rural areas, reduce the redundant construction of older adults care facilities, improve the quality of older adults care service and increase the willingness of older adults to participate in older adults care service, the paper firstly analyzes the accessibility of community older adult care services. Based on the theory of welfare pluralism, it constructs the analysis framework of the impact of the accessibility of community older adult care services on the quality of life of older adults. Then, it uses the survey data of Shaanxi Province and applies ordinal logistic regression method to analyze the urban-rural differences in the impact of accessibility on the quality of life of older adults, and discuss the reasons of the urban-rural differences of accessibility effects from the perspective of different welfare support subjects. Finally, from the aspects of the accessibility of older adult care services, service utilization effect, multi-subject participation in older adult care services, the paper puts forward some suggestions to improve the quality of life of older adults.

### Theory Hypotheses

This study is based on the theory of welfare pluralism. The theory of welfare pluralism originates from the exploration of welfare supply path under the background of western economic stagnation in the 1870s, emphasizes that social sectors outside government should participate in the welfare supply, and advocates the diversification of welfare supply. The theory of welfare pluralism holds that the social welfare that citizens need not only comes from the public sector, but also needs the supply and participation of other social sectors. The participation of the business sector, informal social organizations and other sectors can provide social welfare through a bottom-up approach. Besides, the government functions need to be decomposed into different social subjects from top to bottom, and to form the mixed model of multi-subjects participating in the welfare supply.

In the field of older adult care services, the research on the application of welfare pluralism theory mainly focuses on the division of responsibilities and the provision of welfare. Based on the theory of welfare pluralism, Wang and Qi divided the welfare supply of older adult care services into three main parts of government, market and family [17]. From the perspective of welfare pluralism, Huang et al divided the subject of older adult care services into four subjects of government, social organizations, market and family [18]. Applying the theory of welfare pluralism to community older adult care services, Dai divided supply subjects into four parts of government, profit organizations, non-profit organization, and informal support networks, and pointed out that different service providers played their respective roles in older adult care services [19]. Therefore, according to the theory of welfare pluralism, the social support of community older adult care services mainly involves four subjects of government, social organization, family and neighbors.

Affected by different supply subjects such as government, social organizations, families and neighbors, there are certain regional differences and development differences in urban and rural older adult care services in Shaanxi. From the perspective of regional differences, different living styles, acquaintance culture and community environment can affect the needs for older adult care services [20]. Urban older adult show their needs for activity space, social activities and spiritual comfort, while rural older adult need care facilities, transportation, information and health education. From the perspective of development differences, information technology, care content, care quality and other factors can affect the urban and rural older adult to make different choices [21, 22]. Urban communities generally have sufficient elements such as transportation, information, technology and personnel, and high-quality older adult care service content, and can stimulate older adults’s willingness to participate in older adult care services, while the content and form of older adult care services in rural areas are relatively poor, which inhibits older adults’s demand for older adult care services. Therefore, affected by the supply differences in facility investment, content construction, human capital and market environment during the development of community older adult care services, older adults in different communities have different degrees of dependence on different dimensions of accessibility, which in turn results in the urban-rural heterogeneity of impact of accessibility on the quality of life of older adults. So, we can come up with hypothesis 1 that the impact of the accessibility of community older adult care services on the quality of life of older adults has urban-rural heterogeneity.

Government support can affect the utilization way of community older adult care services from the aspects of older adult care policies, coordination and supervision, and information platforms. Firstly, preferential policies for older adult care services can guide market subjects to invest in the supply of older adult care services [23]. As a quasi-public good, older adult care services should meet essential needs and ensure basic living standards for older adults. In order to encourage market resources and social capitals to flow effectively into the field of older adult care services, Shaanxi’s governments have introduced many preferential policies from the aspects of family bed subsidies, older adult care institution subsidies, older adult restaurant subsidies, and tax incentives for older adult products, which have greatly expanded the resources of older adult care services. Secondly, as the leader of the construction of older adult care services, the government can guide the construction direction and cooperation forms of different subjects such as family, community, market and social organizations [24]. Through policy guidance, standard setting, administrative management, supervision, assessment and other means, the government can standardize the content, methods and ways of different subjects to provide older adult care services, and ensure the high-quality contents of older adult care services. Finally, the government can establish a unified information platform for older adult care services to improve the resources allocation through data analysis [25]. The government, as the main manager, can collect data of different resources such as older adults, enterprises and social organizations, coordinate the resources allocation in different communities according to demand, and improve the timeliness and effectiveness of the resources supply of older adult care service. So, we can come up with hypothesis 2 that government support can strengthen the utilization of older adult care services in rural areas and improve the quality of life of older adults.

Social organization support can improve the utilization ways and forms of community older adult care services by undertaking older adult care services, voluntary services, supervision and management services. First of all, social organizations, as formal non-profit organizations with voluntary and autonomous characteristic, are different from market enterprises and are of a public nature [26]. They can supply older adult care services with public characteristic, such as daily care, emotional solace, restaurants for older adults and other care services. Social organizations can mobilize social forces to participate in older adult care services and improve the accessibility of older adult care services. Secondly, social organizations can provide voluntary services for older adults, including education, propaganda of filial piety, legal aid, caring for older adults and other services [27], timely meet the daily needs of older adults and enhance a sense of belonging. Finally, as a third party, social organizations can supervise the government’s and market’s provision of older adult care services. Social organizations can provide feedback and suggestion on the products, contents and supply forms of older adult care services based on the accessibility and operability of care contents, and improve the service utilization satisfaction of older adults. So, we can come up with hypothesis 3 that social organization support can strengthen the utilization of older adult care services in urban areas and improve the quality of life of older adults.

Family support can improve the utilization satisfaction of community older adult care services from the aspects of family care, emotional support and economic support. First of all, family care can increase the opportunity for older adults to access the care resources [28]. Family members can pick up older adults to community older adult care service centers, senior universities, community health centers and other places. Especially for the device-helping older adult and older adults aged 80 and above, family care can increase their ability and willingness to use older adult care services. Secondly, the emotional support of the family can increase the confidence of older adults to use older adult care services [29]. The help of family members and relatives can help older adults simplify the participation process of older adult care services, explain the usage of older adult care products and facilities, and help older adults participate in older adult care services more quickly. Moreover, children’s companionship and intergenerational support can also increase the satisfaction and sense of acquisition. Finally, family economic support can improve older adults’s ability to consume older adult care services [30]. Older adults have limited economic sources and mainly rely on basic pension and savings. Especially for rural older adults, limited economic income will reduce consumption of older adult care services. Therefore, certain family economic support can alleviate the economic pressure of older adults, improve their consumption of medical services and older adult care products, and improve the quality of life of older adults. So, we can come up with hypothesis 4 that family support can strengthen the utilization of older adult care services in rural areas and improve the quality of life of older adults.

Neighborhood support can improve the utilization effect of community older adult care services through mutual support and social communication. On the one hand, the neighbor’s mutual support can increase the supply of older adult care service resources [31]. In the process of older adult care services, older adults can increase the utilization opportunities of older adult care service through borrowing farm tools, providing idle service resources, sharing service facilities, etc. Especially for rural older adults, they have the basis of stable acquaintance network and mutual support cultural, so different forms of neighborhood support, such as neighborhood care, changing workers and clan older adult care, can increase the supply of older adult care resources. On the other hand, neighborhood communication can increase the sense of acquisition of older adults [32]. Neighborhood communication can reduce the loneliness of older adults through participating in group activities, making up for the emotional lack of older adults due to the lack of child care, and improving the physical and mental health of older adults. Moreover, neighbors can share information to improve the adaptability and acceptability of older adult care services, eliminate older adult’s concerns about the form and content of older adult care services, and improve social participation ability of older adults. So, we can come up with hypothesis 5 that Neighborhood support can strengthen the utilization of older adult care services in rural areas and improve the quality of life of older adults.

## Methods

### Data and Sample

In this paper, the data comes from the social survey of the older adult care services in Shaanxi Province in 2019. The survey team consists of more than 20 people, including teachers, doctoral students and master’s students. This paper adopts the stratified sampling method. First, ten cities in Shaanxi Province are sampled at the first layer, and then Hanzhong, Yan’an and Baoji are sampled at the second layer respectively. For example, Hanzhong City randomly selected Hantai District and Chenggu County as two samples, and then communities and villages in two samples are randomly selected at the third layer. For example, Hantai District extracts Renmin Road community, East Tower Road community and Town Lion village. And Chenggu County extracts East city community, Zhongshan Street community, Guojiashan village and so on. Finally, we randomly investigate older adults in community and village.

Baoji city, located on the border of Shaanxi Province, is the second largest city in Shaanxi Province, with 655,000 older adult aged 60 and above, accounting for 17.3% of the total population. The number of older adult is constantly increasing, and the demand for older adult care services and medical care is constantly rising. Yan’an City, located in northern Shaanxi province, has 15.57 percent of the population aged 60 and above and 10.3 percent of the population aged 65 and above. Hanzhong City is located in the southwest of Shaanxi Province. There are 786,600 older adult aged 60 or above, accounting for 24.31% of the total population, and aged 65 or above accounting for 17.71%. The three regions are distributed in different places in Shaanxi Province and have different levels of economic development, but they all have the characteristics of high aging population and rapid development of older adult care services. Therefore, the three samples of Hanzhong, Baoji and Yan’an are representative to a certain extent in Shaanxi.

A total of 949 pieces of survey data are collected. Based on the principles of accuracy, completeness and applicability of data quality, by checking the relevant information of the questionnaire, we supplement and correct the questionnaire as much as possible, and eliminat the wrong questionnaires and blank questionnaires. Finally, 660 questionnaires are obtained, including 233 in Hanzhong City, 208 in Yan’an City and 219 in Baoji City. After excluding invalid questionnaires, the sample size in each region presents an average distribution, so the target sample has a strong representativeness for the three cities.

Due to limitations such as the survey personnel, survey time, and administrative approval procedures, we only select three cities in Shaanxi Province as the sample representatives. This may have masked the aging situation in some special regions such as ethnic minority areas. Moreover, because rural samples are unevenly distributed across counties, this study also failed to conduct proportional sampling according to different sampling standards such as population distribution, economic differences and geographical differences. This may have masked the differences and particularities of different regions. Although there were the limitations in the sampling representativeness, the sample from the three cities is able to well represent most areas in Shaanxi Province. Therefore, the results presented in this study also mainly focus on analyzing the common problems existing in Shaanxi Province and the corresponding optimization strategies.

### Variable Selection

Dependent variable. The paper takes the quality of life of older adults as the dependent variable. Based on the definition of the quality of life of older adults by the World Health Organization, the paper divides the quality of life of older adults into four aspects of physical condition, psychological status, social relationship and living environmental satisfaction [33]. The physical condition of older adults directly determines older adults’s mobility and life autonomy. Psychological status reflects the satisfaction of older adults with their self-cognition and social status. Social relationship of older adults people with neighborhood, kinship and so on can reflect their sense of social integration and social belonging. Living environmental satisfaction of older adults can reflect the construction situation and service quality of community older adult care services.

Independent variable. This paper takes the accessibility of community older adult care services as the dependent variable, including five dimensions of approachability, availability, acceptability, accommodation and affordability [34].

Control variables. Considering that the individual characteristics of older adults have a great impact on the quality of life of older adults, and referring to the study of scholars Kang [35] and Daengthern et al [36] on factors affecting the quality of life of older adults, we choose age, gender, education, marriage as control variables to ensure that the results are not affected by the personal characteristics of older adults.

We use the Likert method to evaluate indicators, and the options are divided into five levels from weak to strong. The specific characteristics of the indicators are shown in Table 1.

**TABLE 1:** Variables, indicators and sample characteristics (Urban-Rural Differences of Impact of the Accessibility of Community Older Adult Care Services on the Quality of Life of Older Adults, Shaanxi, China. 2019).

Variable	Indicator (mark)	Question	Options	Mean	Median	Standard deviation
Welfare of older adults	Physical condition	How do you rate your physical condition	1. Very bad 2. Bad 3. Average 4. Better 5. Well	3.49	4	1.08
	Psychological status	How do you rate your psychological status	1. Very bad 2. Bad 3. Average 4. Better 5. Well	4.21	4	0.93
	Social relationship	Do you have a good relationship with your neighbors and friends	1. Very bad 2. Bad 3. Average 4. Better 5. Well	4.34	4	0.78
	Environmental satisfaction	How do you rate your living environment of the community	1. Very bad 2. Bad 3. Average 4. Better 5. Well	3.61	4	1.33
Accessibility of community older adult care services	Approachability (*AR*)	Synthetic indicator	​	3.66	3.85	0.91
	Availability (*AV*)	Synthetic indicator	​	2.56	2.47	1.01
	Acceptability (*AT*)	Synthetic indicator	​	3.41	3.58	0.95
	Accommodation (*AD*)	Synthetic indicator	​	3.52	3.65	0.85
	Affordability (*AF*)	Synthetic indicator	​	3.40	3.44	0.65
Mediator variable	Service utilization satisfaction (*ST*)	How do you rate degree of satisfaction of community older adult care services	1. Very bad 2. Bad 3. Average 4. Better 5. Well	3.46	4	1.05
Moderator variable	Government support (*GS*)	How often do government supports community older adult care services	1. Never 2. Seldom 3. Sometimes 4. Often 5. Usually	3.61	4	1.26
	Social organization support (*OS*)	How often do voluntary organizations provide services for older adults	1. Never 2. Seldom 3. Sometimes 4. Often 5. Usually	3.41	3	1.14
	Family support (*FS*)	How often do family members support each other	1. Never 2. Seldom 3. Sometimes 4. Often 5. Usually	4.14	4	0.92
	Neighborhood support (*NS*)	How often do neighborhood help each other	1. Never 2. Seldom 3. Sometimes 4. Often 5. Usually	4.27	4	0.84
Control variables	Personal characteristics	Age (*NL*)	1. 60–65 2. 65–70 3. 70–75 4. 75–80 5. Above 80	-	-	-
		Gender (*GE*)	1. Male 0. Female	-	-	-
		Education (*ED*)	1. Elementary 2. Junior 3. High 4. Junior college 5. Bachelor	1.96	2	1.04
		Marriage (*MR*)	1. Yes 0. Other	0.71	1	0.45

### Measurement of Accessibility

According to the definition of accessibility by Penchansky R and Thomas J [37], we define the accessibility of community older adult care service as older adults’s subjective perception of the matching degree between the supply and demand of community older adult care service resources. Based on the policy content, regional characteristics, construction status and characteristics of older adults care service in Shaanxi Province, we draw up the draft of the indicators system suitable for the accessibility of the community older adult care service in Shaanxi Province, and then invite experts to discuss the draft. The expert group are composed of the authors, 4 teachers, 3 doctors and 3 masters in aging field and discuss the draft for three times. Finally, we form the indicators system, including 5 dimensions and 9 measure indicators. The subjective indicators are measured with Likert method, and older adults score indicators according to their actual feelings.

In order to effectively measure the accessibility of community older adult care services, using the idea of dimension reduction, the paper combines several indicators of accessibility into a single indicator, which is not only conducive to measuring the accessibility, but also conducive to the subsequent analysis of the relationship between accessibility and the quality of life of older adults. Therefore, the paper uses factor analysis method to assign weights to different indicators and forms synthetic indicators of five dimensions of accessibility with SPSS20.0 [38].

Before using factor analysis method, we test the reliability of the data from five dimensions of accessibility, and the Alpha coefficient are more than 0.7, indicating that the questionnaire data are reliable. The Kaiser-Meyer-Olkin values are greater than 0.5, indicating that the correlation between variables was high. The Bartlett tests are significant at the level of 0.000, indicating that the assumption of the independence of variables is not established, so the factor analysis method is suitable.

Then, the objective weight of each index is determined according to the proportion of variance contribution rate of each common factor in the total variance contribution rate and the characteristic value. Since one principal component is extracted from each dimension, the weight of each indicator is equal to the proportion of component value.

Finally, according to the weights of the above indicators, we can calculate the synthetic indicators values of the accessibility dimensions of community older adult care services. The calculation Formulas 1-5 is as follows.

$$AR=0.289A_{1}+0.258A_{2}+0.303A_{3}+0.150A_{4}$$ (1)

$$AV=0.102B_{1}+0.257B_{2}+0.268B_{3}+0.222B_{4}+0.151B_{5}$$ (2)

$$AT=0.074C_{1}+0.220C_{2}+0.227C_{3}+0.175C_{4}+0.224C_{5}+0.080C_{6}$$ (3)

$$AD=0.141D_{1}+0.188D_{2}+0.246D_{3}+0.225D_{4}+0.200D_{5}$$ (4)

$$AF=0.229E_{1}+0.175E_{2}+0.302E_{3}+0.294E_{4}$$ (5)

From the perspective of the mean, median and standard deviation values of the synthetic indicators, we can find that the standard deviation of the synthetic indicator of each dimension of accessibility is close to 1, indicating that the data is concentrated at a certain level and the sample has no outlier. According to the sample data, the mean and median values of approachability, acceptability, accommodation and affordability are at a medium level, indicating that the service content, organizational form and service cost of the older adult care services in Shaanxi Province meet the needs of older adults. The mean and median values of availability are 2.56 and 2.47 respectively, indicating that the availability of older adult care services in the sample is low. The values indicate that the resources supply such as the facility construction, information platform, service personnel and financial support is limited, which is also consistent with the policy concept of paying attention to the inclusive and full coverage of older adult care services in Shaanxi at this stage. On the whole, the sample conforms to the actual situation of Shaanxi’s older adult care service, and the synthetic indicators are reliable.

## Results

### Urban-Rural Differences

Before the discussion on the impact of the accessibility of older adult care services on the quality of life of older adults, the paper firstly adopts the Seemingly Unrelated Regression with Stata15 to test the coefficient differences between the groups. The results shows that the effects of acceptability and affordability on physical Condition are different among groups. The influence of accommodation on social relationship is different among groups. The effects of acceptability, availability and accommodation on environmental satisfaction are different among groups.

In order to explore the urban-rural differences in the impact of accessibility on the quality of life of older adults, the paper divides the sample of older adults into two sub-samples of urban older adults and rural older adults, uses the Ordinal Logistic Regression method and builds the empirical model of the accessibility of older adult care services on the quality of life of older adults [39].

The Ordinal Logistic Regression is a Logit model based on cumulative distribution. Assuming that the dependent variable is an ordered value assigned from 1 to J, the cumulative Logit of the dependent variable $y_{i}\leq j\left|x_{i}\right.$ and $y_{i}>j\left|x_{i}\right.$ can be expressed as its basic theoretical model (6).

$$l_{j}\left(x_{j}\right)=\log\left[\frac{\mathrm{Pr}\left(y_{i}\leq j\left|x_{i}\right.\right)}{\mathrm{Pr}\left(y_{i}>j\left|x_{i}\right.\right)}\right]=\alpha_{j}+\beta X$$ (6)

In Formula 6, X denotes explanatory variables, specifically including core explanatory variables and control variables. β denotes the matrix of coefficients corresponding to X . j denotes the sequence number assigned to the dependent variable from 1 to J . α denotes the intercept term.

The coefficient of each independent variable in coefficient matrix β does not represent the influence of the independent variable on the dependent variable, but the relative proportion of the occurrence of $y_{i}\leq j\left|x_{i}\right.$ and $y_{i}>j\left|x_{i}\right.$ in Formula 6, which is called the Odds ratio. The Odds ratio describes that when the independent variable is increased by one unit, the occurrence ratio of the dependent variable belonging to the lower group is $e^{-\beta }$ times that of the higher group. According to the above settings, the empirical Equation 7 in this paper is as follow.

$$l_{j}\left(x_{j}\right)=\log\left[\frac{\mathrm{Pr}\left(y_{i}\leq j\left|x_{i}\right.\right)}{\mathrm{Pr}\left(y_{i}>j\left|x_{i}\right.\right)}\right]=\alpha_{1}ACC+\alpha_{2}X+\varepsilon$$ (7)

In Formula 7, $l_{j}\left(x_{j}\right)$ denotes dependent variable, that is, the different dimensions of the quality of life of older adults. ACC denotes independent variable, that is, the five dimensions of accessibility. X denotes the control variable, α denotes the regression coefficient, and ε denotes the error term.

Before conducting the ordered regression model, we perform a parallel line test with SPSS20.0. It is found that the P-values of all models are greater than 0.05, indicating that the models accept the null hypothesis and the ordinal logistic regression model can be used. The results of ordinal logistic regression with Stata15 are shown in Table 2.

**TABLE 2:** Results of the Urban-rural Differences of the Impact of Accessibility on the Quality of Life of older adults (Urban-Rural Differences of Impact of the Accessibility of Community Older Adult Care Services on the Quality of Life of Older Adults, Shaanxi, China. 2019).

Variable	Sample of urban older adults				Sample of rural older adults			
	Physical condition	Psychological status	Social relationship	Environmental satisfaction	Physical condition	Psychological status	Social relationship	Environmental satisfaction
*AR*	0.047 (0.470)	0.054 (0.420)	−0.019 (0.781)	0.257*** (0.000)	0.099 (0.331)	0.123 (0.228)	0.038 (0.721)	0.449*** (0.000)
*AT*	0.176** (0.028)	0.230*** (0.006)	0.081 (0.332)	0.315*** (0.000)	0.137 (0.151)	0.229** (0.024)	0.122 (0.230)	0.072 (0.440)
*AF*	0.267** (0.017)	0.086 (0.457)	0.138 (0.240)	0.0569 (0.607)	0.187 (0.112)	0.148 (0.229)	−0.090 (0.478)	−0.071 (0.541)
*AD*	0.077 (0.414)	0.093 (0.333)	0.196** (0.047)	0.286*** (0.005)	−0.106 (0.337)	−0.073 (0.530)	0.185 (0.117)	0.154 (0.179)
*AV*	0.173* (0.055)	0.176* (0.058)	0.069 (0.467)	0.386*** (0.000)	0.148 (0.229)	0.422*** (0.002)	0.189 (0.154)	0.119 (0.339)
*NL*	−0.281*** (0.000)	0.185** (0.024)	0.020 (0.802)	0.412 (0.594)	−0.237** (0.012)	−0.147 (0.138)	−0.219** (0.028)	0.033 (0.725)
*GE*	0.078 (0.707)	0.246 (0.261)	−0.362* (0.099)	0.022 (0.015)	0.369 (0.128)	0.594** (0.022)	0.166 (0.523)	0.116 (0.629)
*MR*	0.305 (0.236)	0.301 (0.264)	−0.150 (0.592)	−0.074 (0.784)	−0.161 (0.535)	0.247 (0.381)	−0.132 (0.648)	0.108 (0.685)
*ED*	0.043 (0.679)	0.031 (0.777)	−0.049 (0.659)	0.070 (0.496)	0.037 (0.818)	0.154 (0.351)	0.129 (0.447)	0.297* (0.061)
LR	52.33	29.08	14.33	92.70	17.09	35.87	20.23	35.38
Log-l	−498.14	−412.66	−366.36	−510.42	−401.35	−317.68	−294.08	−395.27

P-values are in parentheses. *** means significant at the 1% level, ** means significant at the 5% level, and * means significant at the 10% level.

In Table 2, the results show that the availability, acceptability and affordability of urban older adult care services have significant positive impacts on the physical condition of older adults, while the five dimensions of the accessibility of rural older adult care services have no significant impacts on the physical condition of older adults. This may be because the physical exercise of urban older adult depends to some extent on the number, type and use cost of older adult care services. However, due to their long-term participation in farming activities, older adults in rural areas rarely rely on fitness equipment for physical exercise, and most of older adults in rural areas rely on their child care and self care [40], so the impact of older adult care services on physical condition is not significant.

The availability and acceptability of community older adult care services have a significant positive impact on the psychological status of older adults in urban and rural areas, which indicates that the increase of number and the simplification of operability of older adult care facilities, will improve the sense of acquisition of older adults to social capital. This is mainly because the perception of older adults to social capital depends on the perceived usability and sufficiency of older adult care facilities. The more older adult care service resources are supplied, the higher elder people’s happiness is [41]. In addition, diversified older adult care facilities can effectively make up for the lack of family care, increase the social interaction of older adults and enrich the social role of older adults.

The accommodation of community older adult care service has a significant positive impact on the social relationship of urban older adults, while the five dimensions of the accessibility of older adult care services have no significant impact on the social relationship of rural older adults, which indicates that the organization form, supply form and service process of older adult care services have a certain promoting role in shaping the social relationship of urban older adults. This may be due to the fact that in the process of urban older adult care services, collective activities, group mutual assistance and other forms of activities can deepen the understanding between older adults, and expand the social network of older adults [42]. However, the formation of social relationship among older adults in rural areas mostly depends on the local friends and close network of relatives, which has little to do older adult care services. Moreover, the supply forms of older adult care services in rural areas are relatively simple at present, focusing on spontaneous organization and mutual support, so the effect of older adult care services on strengthening social relationship has not yet emerged.

The availability, acceptability and accommodation of community older adult care services have a significant positive impact on the environmental satisfaction of urban older adults, while the impact on the environmental satisfaction of rural older adults is not significant, which indicates that the facilities construction, service process, platform construction and organizational form of urban older adult care services have improved the community environment of residents to a great extent. Compared with cities, older adult care services in rural areas are relatively simple and older adult care facilities are limited [8], which has little impact on improving residents’ environmental satisfaction. However, the approachability of older adult care services has a significantly positive impact on the environmental satisfaction of urban and rural older adult, mainly because the improvement of approachability of older adult care services is conducive to improving residents’ traffic conditions, and convenient travel is an important factor affecting living environmental satisfaction of older adults.

In addition, availability has the greatest impact on quality of life of urban and rural older adult, indicating that the sufficiency of facilities, products and services of older adult care services is the key to improving the quality of life of older adults. Although Shaanxi is speeding up the construction of older adult care services, there is a mismatch between supply and demand of older adult care services resources, mainly due to the following reasons. First, the policy-driven public construction of older adult care services deviates from the real needs of older adults. Second, the market is difficult to meet the needs of older adults for older adult care services [43, 44]. Therefore, government can improve the availability of community older adult care services and the quality of life of older adults by accelerating the public construction of older adult care services, targeting the real needs of older adults, and stimulating the market supply of older adult care services.

## Discussion

In order to analyze the causes of the urban-rural differences of the impact of the accessibility of community older adult care services on the quality of life of older adults, from the perspective of social support, the paper introduces four influential factors of government support, social organization support, family support and neighborhood support to analyzes the mechanism of social support affecting the utilization of older adult care services. And it construct the following regression Equations 8, 9.

$$Y=t_{1}ACC+t_{2}SU+t_{3}X+\varepsilon_{4}$$ (8)

$$Y=c_{1}ACC+c_{2}SU+c_{3}SU\times M+c_{4}X+\varepsilon_{5}$$ (9)

In the equation, SU denotes service utilization utilization. M denotes four factors of social support. c and t denotes regression coefficient.

### Effect of Government Support

We choose the four dimensions of the quality of life of older adults as the dependent variable, and the interaction item between service utilization satisfaction and government support as the core independent variable. The empirical results are shown in Table 3.

**TABLE 3:** The effect of government support on the service utilization satisfaction (Urban-Rural Differences of Impact of the Accessibility of Community Older Adult Care Services on the Quality of Life of Older Adults, Shaanxi, China. 2019).

Variable	Sample of rural older adults				Sample of urban older adults			
	Physical condition	Psychological status	Social relationship	Environmental satisfaction	Physical condition	Psychological status	Social relationship	Environmental satisfaction
*AR*	0.098 (0.339)	0.090 (0.389)	0.026 (0.815)	0.440*** (0.000)	0.018 (0.784)	0.006 (0.928)	−0.072 (0.320)	0.231*** (0.001)
*AT*	−0.430 (0.245)	−0.054 (0.875)	−0.066 (0.853)	0.114 (0.743)	0.953*** (0.000)	0.478** (0.042)	−0.202 (0.426)	0.224 (0.316)
*AF*	0.147 (0.216)	0.164 (0.204)	−0.130 (0.338)	−0.074 (0.528)	0.276** (0.015)	0.086 (0.472)	0.101 (0.412)	0.047 (0.671)
*AD*	−0.139 (0.216)	−0.135 (0.271)	0.155 (0.223)	0.112 (0.340)	0.079 (0.411)	0.015 (0.880)	0.087 (0.400)	0.224** (0.018)
*AV*	0.028 (0.845)	0.222 (0.147)	0.024 (0.880)	0.013 (0.930)	0.186 (0.140)	0.126 (0.336)	0.197 (0.153)	0.293** (0.016)
*ST*	−0.296 (0.389)	0.066 (0.843)	0.067 (0.844)	0.243 (0.472)	0.986*** (0.000)	0.480* (0.093)	−0.498 (0.107)	0.072 (0.790)
*GS*	0.175 (0.172)	0.849*** (0.000)	1.032*** (0.000)	0.074 (0.577)	0.286** (0.011)	0.784*** (0.000)	0.859*** (0.000)	0.201* (0.068)
ST*GS	0.144 (0.118)	0.073 (0.417)	0.047 (0.613)	−0.012 (0.895)	−0.245*** (0.000)	−0.083 (0.249)	0.098 (0.193)	0.027 (0.687)
*NL*	−0.253*** (0.008)	−0.110 (0.287)	−0.194* (0.064)	0.031 (0.739)	−0.299*** (0.000)	0.180** (0.031)	0.008 (0.920)	0.031 (0.686)
*GE*	0.338 (0.165)	0.578** (0.032)	0.085 (0.753)	0.111 (0.644)	0.014 (0.947)	0.230 (0.307)	−0.456** (0.046)	0.025 (0.906)
*MR*	−0.114 (0.665)	0.145 (0.620)	−0.228 (0.451)	0.088 (0.741)	0.203 (0.433)	0.183 (0.516)	−0.123 (0.674)	−0.108 (0.692)
*ED*	0.022 (0.890)	0.074 (0.665)	0.106 (0.547)	0.296* (0.064)	0.070 (0.514)	0.036 (0.750)	−0.060 (0.602)	0.072 (0.483)
LR	23.53	78.90	77.61	37.79	71.40	77.01	69.34	97.35
Log-l	−398.13	−296.17	−265.39	−394.06	−488.61	−388.69	−338.85	−508.10

P-values are in parentheses. *** means significant at the 1% level, ** means significant at the 5% level, and * means significant at the 10% level.

According to the results in Table 3, the interaction item between service utilization satisfaction and government support has a significant negative impact on the physical condition of urban older adults, indicating that government support has reduced the utilization satisfaction of older adult care services. The main reasons are as follows. First, the government’s support policies for older adult care services have raised the expectations of older adults. However, when the actual services fail to meet the promised standards due to insufficient execution efficiency or resource misallocation, it will further reduce the health satisfaction of older adults. Second, government-led older adult care services often aim for standardization and large-scale operation. However, the health needs of older adults are highly heterogeneous. For instance, disabled older adult require professional rehabilitation, while empty-nest older adult need psychological support. When government resources are concentratedly invested in “one-size-fits-all” services, there is a disconnection between individual satisfaction and actual health improvement, which reduces the health satisfaction of older adults. This is consistent with the research conclusion of Kajonius and Kazemi [45], that is, the service object, economic support and service content of older adults care service policy can directly affect the service satisfaction of older adults. He pointed out that the clarification of fiscal expenditure systems such as older adult subsidies, operation subsidies, and facility subsidies could effectively solve the structural contradiction between supply and demand of older adult care services, indicating that government financial support was an important factor in improving older adults’s ability to use older adult care services [46]. Xu and Zhang pointed out that the government could effectively improve the quality of older adult care services by evaluating the quality of service contents and supervising the performance of service personnel, which reflected the importance of government support for the construction of older adult care services and further proved the conclusion of this paper [16].

### Effect of Social Organization Support

We choose the four dimensions of the quality of life of older adults as the dependent variable, and the interaction item between service utilization satisfaction and social organization support as the core independent variable. The empirical results are shown in Table 4.

**TABLE 4:** The effect of social organization support on the service utilization satisfaction (Urban-Rural Differences of Impact of the Accessibility of Community Older Adult Care Services on the Quality of Life of Older Adults, Shaanxi, China. 2019).

Variable	Sample of rural older adults				Sample of urban older adults			
	Physical condition	Psychological status	Social relationship	Environmental satisfaction	Physical condition	Psychological status	Social relationship	Environmental satisfaction
*AR*	0.128 (0.220)	0.125 (0.230)	0.037 (0.730)	0.372*** (0.001)	0.045 (0.500)	0.056 (0.415)	0.006 (0.936)	0.249*** (0.000)
*AT*	0.087 (0.416)	0.284** (0.011)	0.059 (0.595)	0.035 (0.731)	0.143* (0.090)	0.181** (0.041)	0.024 (0.792)	0.284*** (0.001)
*AF*	0.182 (0.119)	0.143 (0.252)	−0.112 (0.385)	−0.123 (0.299)	0.269** (0.017)	0.073 (0.530)	0.143 (0.227)	0.043 (0.698)
*AD*	−0.027 (0.825)	−0.085 (0.521)	0.119 (0.379)	−0.113 (0.379)	0.080 (0.456)	−0.003 (0.981)	0.186 (0.103)	0.082 (0.448)
*AV*	0.080 (0.583)	0.247 (0.110)	−0.011 (0.946)	−0.101 (0.499)	0.115 (0.360)	0.058 (0.649)	0.195 (0.141)	0.225* (0.072)
*ST*	0.131 (0.473)	0.650*** (0.002)	0.228 (0.246)	0.242 (0.223)	−0.031 (0.860)	−0.045 (0.805)	−0.485*** (0.008)	0.113 (0.519)
*OS*	−0.273** (0.029)	−0.142 (0.298)	−0.014 (0.918)	0.586*** (0.000)	−0.053 (0.656)	0.182 (0.130)	−0.008 (0.950)	0.388*** (0.002)
ST*OS	0.041 (0.164)	−0.042 (0.206)	0.044 (0.169)	−0.026 (0.417)	0.035 (0.186)	0.029 (0.316)	0.067** (0.018)	−0.016 (0.576)
*NL*	−0.248*** (0.009)	−0.151 (0.131)	−0.232** (0.021)	−0.007 (0.937)	−0.293*** (0.000)	0.182** (0.026)	0.018 (0.825)	0.043 (0.579)
*GE*	0.390 (0.110)	0.630** (0.017)	0.153 (0.560)	0.189 (0.437)	0.062 (0.768)	0.244 (0.267)	−0.428* (0.054)	0.042 (0.843)
*MR*	−0.108 (0.680)	0.284 (0.317)	−0.067 (0.818)	0.130 (0.627)	0.299 (0.252)	0.341 (0.211)	−0.076 (0.789)	−0.023 (0.934)
*ED*	0.074 (0.642)	0.164 (0.327)	0.154 (0.371)	0.259 (0.109)	0.052 (0.623)	0.035 (0.751)	−0.063 (0.575)	0.069 (0.507)
LR	26.03	47.47	28.4	57.69	54.68	33.37	0.0336	104.05
Log-l	−396.88	−311.88	−289.99	−384.11	−496.96	−410.51	−362.34	−504.75

P-values are in parentheses. *** means significant at the 1% level, ** means significant at the 5% level, and * means significant at the 10% level.

As shown in Table 4, the interaction term between service utilization satisfaction and social organization support has a significant impact on the social relationship of urban older adults, which reflects that social organization, as an important supplier of older adult care services, can improve the service utilization of older adults from the aspects of older adult care facilities, spiritual comfort and social activities. The social organization support can strengthen the influence of service utilization on the social relationship of urban older adults, mainly because social organization can directly guide and assist older adults to participate in older adult care services activities. Older adult care service activities can broaden the social network of older adults, shape the different social roles and social responsibilities of older adults, and then improve the social relationship of older adults. However, the impact of the interaction term on the social relationship of older adults in rural areas is not significant, mainly because rural areas already have familiar and close social networks, and the participation of social organizations has little impact on the improvement of social relations among older adults. The impact of social organizations on rural older adults care service is more reflected in the construction of facilities, resource supply and other aspects [47].

### Effect of Family Support

We choose the four dimensions of the quality of life of older adults as the dependent variable, and the interaction item between service utilization satisfaction and family support as the core independent variable. The empirical results are shown in Table 5.

**TABLE 5:** The effect of family support on the service utilization satisfaction (Urban-Rural Differences of Impact of the Accessibility of Community Older Adult Care Services on the Quality of Life of Older Adults, Shaanxi, China. 2019).

Variable	Sample of rural older adults				Sample of urban older adults			
	Physical condition	Psychological status	Social relationship	Environmental satisfaction	Physical condition	Psychological status	Social relationship	Environmental satisfaction
*AR*	0.132 (0.207)	0.128 (0.225)	0.043 (0.698)	0.375*** (0.000)	0.029 (0.668)	0.016 (0.819)	−0.061 (0.397)	0.245*** (0.000)
*AT*	0.154 (0.109)	0.247** (0.017)	0.139 (0.195)	0.008 (0.935)	0.178** (0.028)	0.231*** (0.007)	0.113 (0.195)	0.274*** (0.001)
*AF*	0.188 (0.109)	0.170 (0.183)	−0.122 (0.361)	−0.122 (0.300)	0.259** (0.021)	0.072 (0.546)	0.118 (0.335)	0.045 (0.686)
*AD*	−0.020 (0.869)	−0.055 (0.684)	0.190 (0.175)	−0.118 (0.357)	0.077 (0.476)	−0.061 (0.585)	0.150 (0.193)	0.072 (0.507)
*AV*	0.100 (0.493)	0.250 (0.108)	0.022 (0.891)	−0.108 (0.472)	0.116 (0.357)	0.051 (0.698)	0.226 (0.101)	0.222* (0.075)
*ST*	0.137 (0.557)	−0.480* (0.055)	−0.767*** (0.003)	0.064 (0.795)	−0.116 (0.546)	−0.859*** (0.000)	−1.183*** (0.000)	−0.087 (0.638)
*FS*	−0.273** (0.029)	−0.181 (0.196)	−0.072 (0.610)	0.579*** (0.000)	−0.054 (0.648)	0.133 (0.281)	−0.087 (0.495)	0.369*** (0.003)
ST*FS	0.028 (0.435)	0.209*** (0.000)	0.253*** (0.000)	0.016 (0.687)	0.057* (0.081)	0.239*** (0.000)	0.243*** (0.000)	0.036 (0.270)
*NL*	−0.239** (0.012)	−0.102 (0.318)	−0.194* (0.060)	−0.007 (0.944)	−0.294*** (0.000)	0.182** (0.030)	0.009 (0.912)	0.038 (0.627)
*GE*	0.394 (0.106)	0.575** (0.032)	0.080 (0.765)	0.171 (0.484)	0.083 (0.692)	0.277 (0.220)	−0.471** (0.038)	0.029 (0.891)
*MR*	−0.145 (0.579)	0.202 (0.486)	−0.171 (0.570)	0.130 (0.628)	0.284 (0.275)	0.342 (0.224)	−0.119 (0.681)	−0.022 (0.935)
*ED*	0.062 (0.696)	0.144 (0.395)	0.152 (0.387)	0.264 (0.103)	0.058 (0.586)	0.033 (0.769)	−0.061 (0.594)	0.062 (0.552)
LR	24.70	71.35	64.18	57.19	56.01	79.2	63.92	104.95
Log-l	−397.55	−299.94	−272.11	−384.36	−496.30	−387.60	−341.56	−504.30

P-values are in parentheses. *** means significant at the 1% level, ** means significant at the 5% level, and * means significant at the 10% level.

As shown in Table 5, the interaction term of service utilization satisfaction and family support has a significant impact on the psychological status and social relationship of urban and rural older adult, but only have a significant positive effect on the physical condition of urban older adult, indicating that urban older adult pay more attention to the quality of older adult care services rather than the number of facilities. Family support can strengthen the influence of service utilization on the physical condition of older adults, mainly because family support can assist older adults to simplify the participation process of older adult care service, improve the economic ability, adaptability and capability of older adults, and thus increase the opportunities and willingness of older adults to use older adult care service. For example, when accompanied by family members, older adults are more confident in using different types of fitness equipment. With the family support, older adults are also more willing to participate in various activities, which can improve the physical condition of older adults [48].

### Effect of Neighborhood Support

We choose the four dimensions of the quality of life of older adults as the dependent variable, and the interaction item between service utilization satisfaction and neighborhood support as the core independent variable. The empirical results are shown in Table 6.

**TABLE 6:** The effect of neighborhood support on the service utilization satisfaction (Urban-Rural Differences of Impact of the Accessibility of Community Older Adult Care Services on the Quality of Life of Older Adults, Shaanxi, China. 2019).

Variable	Sample of rural older adults				Sample of urban older adults			
	Physical condition	Psychological status	Social relationship	Eenvironmental satisfaction	Physical condition	Psychological status	Social relationship	Environmental satisfaction
*AR*	0.126 (0.225)	0.095 (0.374)	−0.015 (0.902)	0.370*** (0.001)	0.039 (0.562)	0.048 (0.486)	−0.019 (0.805)	0.253*** (0.000)
*AT*	0.147 (0.128)	0.206** (0.045)	0.106 (0.364)	0.007 (0.938)	0.176** (0.030)	0.250*** (0.004)	0.166* (0.078)	0.274*** (0.001)
*AF*	0.199* (0.090)	0.196 (0.129)	−0.069 (0.641)	−0.113 (0.341)	0.258** (0.023)	0.015 (0.903)	0.019 (0.887)	0.025 (0.822)
*AD*	−0.022 (0.857)	−0.091 (0.498)	0.206 (0.170)	−0.118 (0.355)	0.082 (0.445)	−0.030 (0.791)	0.158 (0.197)	0.070 (0.520)
*AV*	0.090 (0.535)	0.218 (0.162)	0.069 (0.685)	−0.104 (0.487)	0.118 (0.350)	0.034 (0.798)	0.145 (0.331)	0.212* (0.091)
*ST*	−0.075 (0.756)	−0.477* (0.062)	−2.224*** (0.000)	−0.035 (0.887)	−0.011 (0.955)	−0.929*** (0.000)	−2.614*** (0.000)	−0.143 (0.452)
*NS*	−0.265** (0.034)	−0.135 (0.331)	−0.008 (0.959)	0.583*** (0.000)	−0.055 (0.646)	0.079 (0.520)	−0.284** (0.042)	0.351*** (0.005)
ST*NS	0.070* (0.067)	0.205*** (0.000)	0.570*** (0.000)	0.034 (0.366)	0.029 (0.389)	0.246*** (0.000)	0.597*** (0.000)	0.050 (0.143)
*NL*	−0.233** (0.015)	−0.121 (0.240)	−0.178 (0.114)	−0.005 (0.955)	−0.284*** (0.000)	0.223*** (0.008)	0.079 (0.387)	0.046 (0.555)
*GE*	0.323 (0.192)	0.408 (0.133)	−0.355 (0.246)	0.139 (0.573)	0.083 (0.690)	0.287 (0.198)	−0.603** (0.015)	0.032 (0.879)
*MR*	−0.157 (0.549)	0.254 (0.378)	−0.082 (0.798)	0.138 (0.605)	0.302 (0.250)	0.541* (0.054)	0.307 (0.332)	0.010 (0.971)
*ED*	0.048 (0.765)	0.104 (0.538)	−0.006 (0.977)	0.251 (0.122)	0.053 (0.619)	0.036 (0.747)	−0.084 (0.495)	0.068 (0.515)
LR	27.47	69.96	164.75	57.84	53.68	76.06	192.48	105.89
Log-l	−396.16	−300.63	−221.82	−384.03	−497.46	192.48	−277.28	−503.83

P-values are in parentheses. *** means significant at the 1% level, ** means significant at the 5% level, and * means significant at the 10% level.

As shown in Table 6, the interaction item of service utilization satisfaction and neighborhood support has a significant impact on the psychological status and social relationship of the urban and rural older adult, but neighborhood support only has a positive impact on the environmental satisfaction of urban older adults, which indicates that neighborhood support can strengthen urban older adults’s service utilization satisfaction and increase their living environment satisfaction. This is mainly due to the fact that harmonious neighborhood relationship can improve the atmosphere of respecting and loving older adults in the community, thus increasing the sense of belonging and happiness of older adults to the community environment. Li also found that social support from friends, neighbors and others had a positive impact on the life satisfaction of rural older adult, and pointed out that social support could increase the social participation behavior of older adults and reduce loneliness [49], which is consistent with the conclusion of the paper. Moghadam et al. pointed out that informal social support such as social communication from relatives and friends could significantly reduce the death anxiety of older adults, and social interaction could effectively improve the wellbeing of life of older adults [50], which also proved that emotional assistance between neighbors was an important factor affecting the quality of life of older adults, which is consistent with the conclusion of the paper.

### Conclusion

Based on the concept of accessibility of community older adult care services, the paper takes the survey data of Shaanxi as an example, and constructs an accessibility indicators system suitable for Shaanxi’s community older adult care services, including the traffic distance, the number of facilities, the service effect, the service cost and other factors of older adult care services. Then through empirical analysis, the paper finds that, firstly, the urban-rural differences in the impact of the accessibility of community older adult care services on the quality of life of older adults are mainly reflected in accommodation and affordability, which is mainly related to the difference of contents, forms and cost of older adult care service in urban and rural areas. Secondly, government support and social organization support are the main reasons for the urban-rural differences of the impact of the accessibility of older adult care services on the quality of life of older adults. Government support can effectively improve the environmental satisfaction of rural older adults by improving infrastructure and optimizing the community environment, and social organization support can improve the social relationship of urban older adults by enriching the forms of older adult care services and shaping the social roles of older adults.

## Data Availability

The datasets used and analyzed in this study are provided by the Questionnaire on Accessibility of Community Older adult care services. The questionnaire was developed for this study and does not involve any personal privacy information. The datasets are available from the corresponding authors on reasonable request.
